# Docosahexaenoic Acid (DHA) and Eicosapentaenoic Acid (EPA)—Should They Be Mandatory Supplements in Pregnancy?

**DOI:** 10.3390/biomedicines12071471

**Published:** 2024-07-03

**Authors:** Mihaela Amza, Bashar Haj Hamoud, Romina-Marina Sima, Mihai-Daniel Dinu, Gabriel-Petre Gorecki, Mihai Popescu, Nicolae Gică, Mircea-Octavian Poenaru, Liana Pleș

**Affiliations:** 1 Department of Obstetrics and Gynecology, “Carol Davila” University of Medicine and Pharmacy, 020021 Bucharest, Romania; mihaela.amza@umfcd.ro (M.A.); gica.nicolae@umfcd.ro (N.G.); mircea.poenaru@umfcd.ro (M.-O.P.); liana.ples@umfcd.ro (L.P.); 2 “Bucur” Maternity, Saint John Hospital, 012361 Bucharest, Romania; 3 Department PhD, IOSUD, “Carol Davila” University of Medicine and Pharmacy, 020021 Bucharest, Romania; mihai-daniel.dinu@drd.umfcd.ro; 4 Department for Gynecology, Obstetrics and Reproductive Medicine, Saarland University Hospital, Kirrberger Straße 100, Building 9, 66421 Homburg, Germany; bashar.hajhamoud@uks.eu; 5 Faculty of Medicine, Titu Maiorescu University, 040441 Bucharest, Romania; gabriel.gorecki@prof.utm.ro; 6 Department of Anaesthesia and Intensive Care, “Carol Davila” University of Medicine and Pharmacy, 020021 Bucharest, Romania; mihai.popescu@umfcd.ro; 7 Department of Anaesthesia and Intensive Care, Bucharest University Emergency Hospital, 050098 Bucharest, Romania; 8 Filantropia Clinical Hospital Bucharest, 011132 Bucharest, Romania

**Keywords:** docosahexaenoic acid, eicosapentaenoic acid, neurological development, visual acuity, postpartum depression, preterm birth

## Abstract

Docosahexaenoic acid (DHA) and eicosapentaenoic acid (EPA) are essential fatty acids for the human body. Seafood and microalgae are the most important sources of omega-3 fatty acids. Supplementation with 200 mg/day of DHA during pregnancy and breastfeeding has been suggested for women and infants in countries with low seafood consumption. Maternal concentration of DHA and EPA was associated with concentration in cord blood and breast milk. High concentrations of DHA and EPA were identified at the level of retinal photoreceptors and neuronal cell membranes. It was observed that supplementation with DHA and EPA during pregnancy had beneficial effects on the neurological development of the fetus and infant by improving language, memory, attention, and hand coordination, affecting sleep patterns, and improving visual acuity. Beneficial effects on the development of the infant were also associated with the maternal intake of omega-3 fatty acids during breastfeeding. Supplementation with DHA and EPA may reduce the risk of preterm birth but also of preeclampsia in low-risk pregnancies. Women of childbearing age should have an intake of 250 mg/day of DHA + EPA from their diet or supplements. To reduce the risk of premature birth, pregnant women must additionally receive at least 100–200 mg of DHA every day. It is recommended that supplementation with omega-3 fatty acids starts before 20 weeks of pregnancy. Beneficial effects on the mother have been identified, such as the reduction of postpartum depression symptoms, the decrease of cardiovascular risk, and the anti-inflammatory role.

## 1. Introduction

Pregnancy represents a period when expectant mothers are focused on their diet, and they try to improve it to ensure the necessary nutrients for the fetus. It is known that certain nutrient deficiencies can be associated with numerous malformations or fetal anomalies. The use of supplements during pregnancy is a frequent practice. In addition to vitamins and minerals, it was found that essential fatty acids have a role in the normal development of the fetus [1]. We conducted a narrative review to describe the effects of omega-3 supplementation during pregnancy.

Omega-3 and omega-6 essential fatty acids were discovered in 1929 by G.O. Burr and M.M. Burr and became a topic of interest for researchers because they were found in high concentrations within cell membranes [2,3]. Polyunsaturated fatty acids such as omega-3 and omega-6 are essential fatty acids with very important roles for good health but which cannot be synthesized de novo by the body. A typical diet is rich in omega-6 fatty acids found in vegetable oils, which contain linoleic acid (LA). It is converted into arachidonic acid through a multistep pathway. Linoleic acid is converted into gamma-linolenic acid (GLA) by the delta-6 desaturase enzyme. GLA is then metabolized into dihomo-γ-linolenic acid (DGLA), which is a precursor of arachidonic acid [4]. This is essential in the development of the fetal central nervous system. A small amount of corn oil, about a teaspoon, is enough to ensure the necessary daily intake of omega-6 [5]. Regarding the fatty acids in the current typical Western diet, there is an omega-6/omega-3 ratio of 20:1 in favor of omega-6 fatty acids that promote inflammation and predispose to numerous diseases. It was considered that in Paleolithic times, when the human species presented an evolution, this ratio was 4:1. The intake of DHA (docosahexaenoic acid) and EPA (eicosapentaenoic acid) is currently about 100–200 mg/day, much lower than in Paleolithic times [6].

Seafood is the best source of DHA and EPA. It was recommended that a pregnant woman consume 8–12 ounces of fish-based food. This ensures an intake of 300–900 mg per day of the most important omega-3 fatty acids, depending on the type of fish. But very few pregnant women have such a diet [7]. However, it was not recommended to consume fish in excess during pregnancy because fatty fish may contain contaminants such as dioxin or similar products that may have negative effects on the development of the fetus [8,9]. In addition, seafood is an important source of microplastics, and their effects on the human body are difficult to assess [10]. The consumption of fish-based preparations can represent a good source of essential trace elements such as zinc and iron, but at the same time, they contain variable concentrations of toxic elements (e.g., mercury, lead, and cadmium) [11]. If the consumption of fish exceeds the limit of two portions per week (approximately 340 g), organic mercury and other toxins contained in seafood can become dangerous [12]. Using supplements based on fish oil reduces this danger because they are purified to eliminate toxins. Another advantage of the supplements was the fact that they contain small amounts of mercury [13,14].

A study included 11,875 women and evaluated diet during pregnancy using a questionnaire. It was observed that there were positive effects on the child’s development if the maternal consumption was more than 340 g of seafood. This study concluded that there was a greater risk of lack of nutrients through the indications of limiting the diet in terms of eating seafood than the negative effects that the excess of this type of food could bring. Low concentrations of DHA were associated with reduced dendritic arborization and disruption of the expression of genes involved in connectivity, neurotransmission, and neurogenesis [15].

Microalgae oil is one of the main sources of DHA. It was observed that DHA from microalgae oil was more easily absorbed and metabolized compared to DHA from fish oil [10]. Microalgae oil is used on a large scale in the food industry and offers DHA with high bioavailability. *Schizochytrium* sp. has become a very important microorganism in the DHA production industry because it is easy and quick to harvest, and it was found that it contains large amounts of DHA that can be easily and efficiently extracted [16]. Krill is another important source of omega-3 fatty acids and is a small shrimp-like crustacean. Its catch from the Atlantic Ocean was simple but was limited to prevent the destruction of the marine ecosystem. It contains large amounts of DHA and EPA because microalgae are a main source of its nutrition [17].

It was observed that the intake of DHA among women from industrialized countries was low [12]. In Western countries, the average intake of DHA varies between 70 and 200 mg/day, and the optimal requirement for mother and fetus was not ensured. DHA supplementation had effects on maternal and fetal outcomes: visual and neurological development, infant growth, and maternal depression. In large-scale studies, it was reported that supplementation with high doses of marine oil was safe in pregnancy [18].

EPA and DHA are the most biologically active omega-3 fatty acids and have numerous positive effects on fetal growth [19,20].

**Figure 1:**
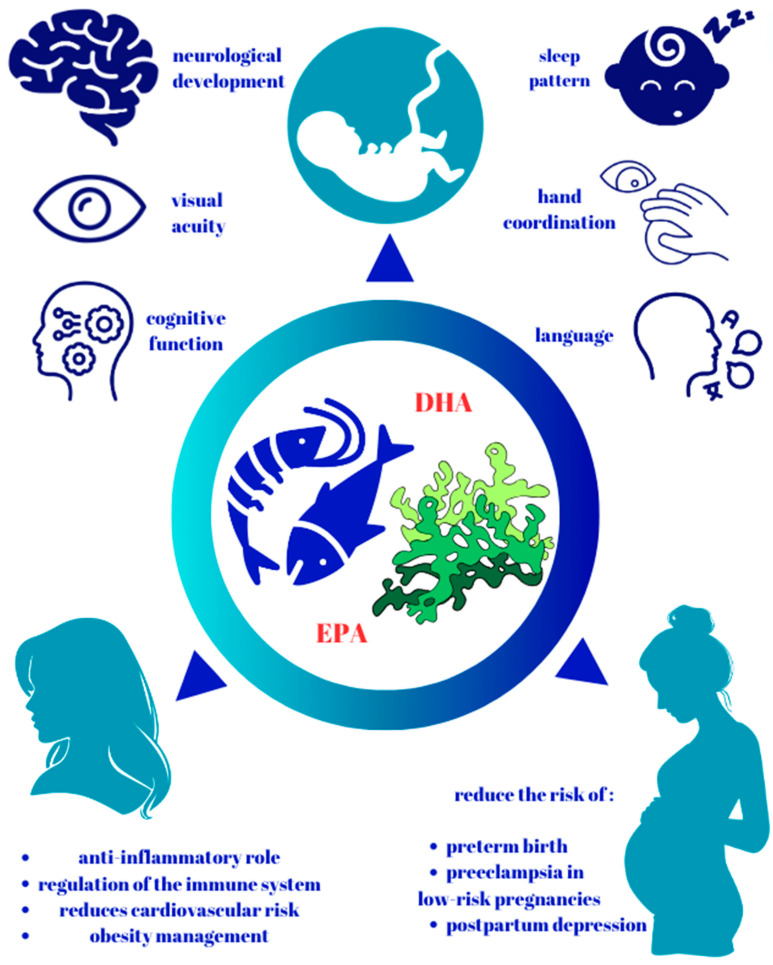
The beneficial effects of supplementing with omega-3 fatty acids during pregnancy. (DHA—docosahexaenoic acid; EPA—eicosapentaenoic acid).

Figure 1 summarizes the beneficial effects of supplementing with omega-3 fatty acids during pregnancy on the development of the fetus and the infant and on the mother, but also on the complications associated with pregnancy.

It was noticed that supplementation with DHA and EPA during pregnancy had beneficial effects on visual acuity [21] and neurological development of the fetus [22] through involvement in the processes of neurogenesis, neuronal signaling, and synaptic plasticity [23]. Supplementation with DHA and EPA could reduce the risk of preterm birth by reducing inflammation, which is a final process in preterm birth [24].

Additionally, supplementation with DHA and EPA can reduce the risk of preeclampsia by decreasing oxidative stress and inflammation of the placenta [25]. It has been observed that supplementation with omega-3 fatty acids can reduce the risk of postpartum depression [26] through neuroprotective and anti-inflammatory mechanisms [27].

One of the positive effects on women is also that omega-3 fatty acids can reduce cardiovascular risk and improve outcomes through various mechanisms, including membrane stabilization, triglyceride lowering, and anti-inflammatory, antiarrhythmic, or antithrombotic properties [28].

## 2. Metabolism, Absorption, and Bioavailability

The precursor of omega-3 fatty acids is α-linolenic (ALA) acid, which can be converted into EPA and then into DHA, and these are more biologically active forms. This process is limited in the human body because the conversion of ALA to EPA competes for the same enzyme with the conversion of linoleic acid to arachidonic acid. Linoleic acid represents the precursor of omega-6 fatty acids [29]. It was found that increasing the intake of ALA in pregnant women does not cause an increase in DHA in the maternal or fetal circulation [30,31]. Therefore, the use of supplements based on alpha-linolenic acid was not encouraged, especially since the body is not able to efficiently convert EPA into DHA [32]. In conclusion, to increase the plasma concentration of DHA and EPA, it is necessary to absorb them from the diet or supplements. The solubility of omega-3 fatty acids is low, and as a result, their bioavailability is affected. The lipid structure in which they are released is very important. Most of the time, supplement formulas are limited by poor lipid digestion, increased dispersion, and moderate solubility. Taking supplements during high-fat meals can improve the absorption of omega-3 fatty acids. It was observed that preparations emulsified with EPA and DHA have a high absorption [33].

Omega-3 index represents one of the most important coefficients used in the evaluation of the bioavailability of omega-3 fatty acids in the body. It was defined as the proportion of DHA and EPA from the total of fatty acids present in erythrocytes. Its value is a percentage. Since erythrocytes have a long life, this parameter indicates long-term bioavailability. Omega-3 fatty acids are susceptible to the oxidation process, and storing them in optimal amounts is essential to limit this process [34]. Limits have been reported for the value of the omega-3 index, and it is desirable to have a value greater than 8%. A moderate value means between 6% and 8%, low is 4–6%, and very low is below 4% [35].

In adults, the DHA consumption of the brain is equivalent to the incorporation rate of 3.8 ± 1.7 mg/day, and the half-life of DHA in the brain is 2.5 years [36]. If DHA disappears from the plasma for 49 days, a 5% decrease in the DHA level in the brain will be found. In a few months, in the case of diets low in omega-3 fatty acids, functional changes may occur in the brain [36].

However, the transfer of DHA from mother to fetus can be altered by various maternal pathologies such as gestational diabetes mellitus [37] and preeclampsia [38]. It was found that maternal habits can influence the effects of DHA supplements. In a study by Gould et al., smoking canceled the beneficial effects of omega-3 fatty acid administration in pregnancy, and non-smoking women had a lower risk of premature birth [39].

Studies concluded that the administration of 200 mg/day of DHA during pregnancy and breastfeeding was sufficient to ensure the necessary amount of this omega-3 fatty acid for women and infants in countries with low seafood consumption [40,41,42].

## 3. Effects on Fetal and Infant Neurodevelopment

Approximately 60% of the dry weight of the brain is represented by fatty acids, especially omega-3 fatty acids, and the most important is DHA [38]. DHA represents approximately 14% of the fatty acids in the brain, while EPA represents only 1% of the total fatty acids at this level [43]. Cell membranes in the gray matter of the brain are enriched with DHA, during fetal development. In the third trimester, the amounts of DHA that accumulate in the central nervous system increase greatly compared to those of other fatty acids. DHA was involved in neurotransmission and gene expression. It was observed that a decrease in the level of DHA causes a decrease in the size of the neurons in certain areas of the brain and, therefore, affects their function [38]. Other effects of DHA on the development of the nervous system were also reported, such as reducing the apoptosis of neuronal cells, protecting cells from oxidative stress, regulating nerve growth factors and ion channels, being involved in serotoninergic and dopaminergic transmission, and determining synaptic growth cones [44].

DHA was involved in numerous neurophysiological functions such as neurogenesis, cellular signaling, cell survival, and neuroinflammation. It has a protective role for the integrity of the blood-brain barrier. It is important that the metabolism of DHA in the brain not be disturbed because neurological or psychiatric conditions may appear [45].

Numerous studies were directed to evaluate the benefits on the fetus and child’s neurological development secondary to DHA supplementation during pregnancy. The data are summarized and presented in Table 1.

A systematic review published by Sherzai et al. in 2023 assessed the impact of omega-3 fatty acid consumption on human neurodevelopment in three periods: in-utero, lactation or infancy, and childhood or adolescence. Short-term benefits were observed in all these periods in terms of working memory, communication, executive function, and visual attention [46].

In a study by Helland et al., it was demonstrated that the administration of supplements containing DHA and EPA during pregnancy and breastfeeding was significantly correlated with the mental performance of children at 4 years [47].

**Table 1:** DHA and EPA supplementation during pregnancy and the child’s neurological development.

Authors (Year)	Participants	Starting Time (Weeks of Pregnancy)	Dose Per Day	Effects
Colombo et al. (2016) [48]	350	14	600 mg DHA No EPA and ALA (α-linolenic acid)	- Increased level of sustained attention in the first year of life - Reduced exhaustion on habituation tasks at 6 and 9 months
Colombo et al. (2019) [49]	350	14	600 mg DHA No EPA and ALA	- Reduced early preterm birth - Benefit in visual attention in infancy
Ramakrishnan et al. (2016) [50]	1094	18–20	400 mg DHA No EPA and ALA	Improved sustained attention in 5-year-old children
Dunstan et al. (2008) [51]	98	20	2200 mg DHA 1100 mg EPA No ALA	Improvement in the child’s eye and hand coordination in 2-year-old children
Ostadrahimi et al. (2018) [52]	150	20	120 mg DHA 180 mg EPA No ALA	Benefits for the communication domain in 4-month-old infants
Campoy et al. (2011) [53]	154	20	500 mg DHA 150 mg EPA No ALA	Maternal DHA level may be associated with later cognitive function in children
Judge et al. (2007) [54]	29	24	300 mg DHA No EPA and ALA	Benefits for infants aged 9 months in problem-solving
Sousa et al. (2023) [55]	60	22–24	1440 mg DHA No EPA and ALA	Beneficial effect on routines/child behavior
Judge et al. (2012) [56]	48	24	300 mg DHA No EPA and ALA	Benefits for infant sleep organization

A randomized study published by Escolano-Margarit et al. included 315 pregnant women who were divided into four groups and who received, starting from 20 weeks of gestation, one sachet per day with 500 mg DHA + 150 mg EPA, or 400 µg/d 5-methyltetrahydrofolate (5-MTHF), or both, or a placebo. This study did not show long-term beneficial or harmful effects of omega-3 fatty acid supplementation during pregnancy on children’s neurological development. By evaluating maternal and fetal levels of DHA, it was found that their higher values during pregnancy were correlated with good performance of children at 5.5 years old in neurological tests [57].

There are also contradictory data in the literature regarding the benefits of consuming omega-3 fatty acids in pregnancy. One of the largest studies on the beneficial effects of DHA supplementation in pregnancy was published by Makrides et al. in 2010 in JAMA. This multicenter, double-blind, randomized trial study included 2399 women pregnant with singletons who were divided into two groups: one receiving daily 800 mg of DHA and 100 mg EPA, and one received capsules without DHA or EPA during pregnancy. A total of 694 children were evaluated at 18 months of age, and the average for the composite cognitive and composite language scores was not different in these two groups. In addition, symptoms related to depression were evaluated among 2320 women in the first 6 months postpartum, and no difference was found between the two groups [58].

The impact of the administration of omega-3 fatty acids (1020 mg DHA, 180 mg EPA, and 9 mg Vitamin E) during pregnancy on neurodevelopment at preschool age (at 4 and 5 years old) was also evaluated, but there were no clear results regarding its benefits [59].

High concentrations of plasma DHA in the mother at parturition influence the sleep pattern in infants in the first days of life. Thus, a lower ratio of active sleep to quiet sleep was found. This fact may suggest a higher maturity of the central nervous system if the mother had high concentrations of plasma DHA [60].

The relationship between the intake of omega-3 fatty acids during pregnancy and the risk of autism spectrum disorders (ASD) in children was evaluated. It has been reported that high maternal concentrations of omega-3 fatty acids in the second half of pregnancy could reduce the risk of ASD in children by up to 40% [61]. The results of another study by Makrides et al. showed that there was no difference regarding the diagnosis of ASD or hyperactivity disorders between the two analyzed groups (DHA supplementation and placebo) [62].

## 4. Effects on the Visual Development

DHA is found in high concentrations both in the membranes of nerve cells and in the photoreceptors of the retina [63]. It was observed that the intake of DHA during pregnancy had beneficial effects on the optimal development of the retina and on the visual acuity of infants [64]. A positive correlation between DHA concentration in fetal blood and visual development in the first year of life has been reported [65]. The results of another study conducted by Jacques et al. showed the beneficial effects of omega-3 fatty acid intake during pregnancy on visual function in school-age children. It has been observed that DHA is important in the development and functioning of the parvocellular pathway [66]. Contradictory data have been reported in the literature. Hurtado et al. found no clear beneficial effects of omega-3 fatty acids on the visual, psychomotor, or cognitive development of infants [67]. In a study by Smithers et al., it was concluded that the intake of 800 mg of DHA during pregnancy did not improve the visual acuity of children at 4 months of age [68]. It was reported that DHA levels in breast milk had effects on visual acuity [69].

## 5. Omega-3 Fatty Acids and Preterm Birth

A series of risk factors for preterm birth (PTB; <37 weeks) and early preterm birth was described, and they had an impact in terms of clinical outcomes but also an economic impact [70,71]. It was observed that a higher maternal supply of omega-3 fatty acids reduced the risk of PTB and early PTB [72]. The analysis of data from the ORIP randomized trial showed that supplementation with omega-3 fatty acids (daily dose of 800 mg DHA + 100 mg EPA) reduces the risk of PTB only in the subgroup of singleton pregnancy and poor status of omega-3 fatty acids at the beginning of pregnancy [73]. It was observed that the intake of omega-3 fatty acids (800 mg DHA + 100 mg EPA) during pregnancy can increase the average duration of pregnancy by a few days and reduce especially the risk of early PTB (<34 weeks) [58,74]. A slight increase in birth weight was also observed [75]. In a meta-analysis by Horvath et al., the only beneficial effect of omega-3 supplementation (daily dose of 900–1080 mg DHA) during pregnancy was to reduce the risk of preterm birth [76].

Cervical inflammation or intra-amniotic infection has been associated with the initiation of labor through the increase of pro-inflammatory cytokines. Activation of fetal T-cells was identified as another trigger for preterm labor [77]. The anti-inflammatory effect of DHA and EPA was described [78]. The production of anti-inflammatory cytokines from the metabolism of DHA and EPA interferes with the production of pro-inflammatory cytokines from the metabolism of omega-6 fatty acids. The synthesis of prostaglandins E2 and F2α is thus affected. These prostaglandins have known effects on the initiation of uterine contractions and cervical ripening [79].

In an expert review published in 2024 that analyzed the relationship between the maternal status of omega-3 fatty acids and the risk of PTB, it was recommended that women of childbearing age should have an intake of 250 mg/day of DHA + EPA from their diet or supplements, and pregnant women should additionally receive supplements with at least 100–200 mg of DHA every day. It was recommended to identify pregnant women with a deficiency of omega-3 fatty acids either through dietary questions or through blood samples, and it was indicated that they receive supplements with 600–1000 mg/day of omega-3 fatty acids (DHA + EPA) or DHA alone. It was recommended that supplementation with DHA start in the second trimester of pregnancy but no later than 20 weeks of pregnancy [80].

## 6. Omega-3 Fatty Acids and Preeclampsia

Hypertensive disorders in pregnancy are health problems with an increasing incidence that can have devastating effects on both the fetus and the mother. Despite the available drugs, there are situations when the only effective therapeutic option is cesarean delivery [81].

It was noticed that the low intake of omega-3 fatty acids was associated with the occurrence of preeclampsia. Omega-3 fatty acids did not only play a role in the second half of pregnancy, they were also involved in implantation and placentation. The effects of DHA supplementation in pregnancy were evaluated, but there were no significant results in the prevention of preeclampsia [82]. The DOMInO (DHA to Optimize Mother Infant Outcome) trial included 2399 pregnant women who were randomly assigned to receive 800 mg/day of DHA starting before 21 weeks of pregnancy and until birth. It was observed that DHA supplementation did not reduce the risk of preeclampsia or gestational diabetes mellitus [83]. The results of a systematic review carried out by Horvath et al. showed that there are no clear enough data to indicate the administration of omega-3 fatty acids in high-risk pregnancies to reduce the risk of intrauterine growth restriction or hypertensive disorders in pregnancy [77].

The effects of DHA were evaluated in a culture of trophoblastic cells, and it was found that it causes cell proliferation and the formation and growth of tubes used as a marker of angiogenesis and stimulates the expression of the gene for VEGF (vascular endothelial growth factor), which was an important factor for angiogenesis placental [84]. Among the effects of DHA involved in the formation and proper functioning of the placenta were also the anti-inflammatory effect and the reduction of oxidative stress [85].

The role of omega-3 fatty acid supplementation during pregnancy to reduce the risk of preeclampsia remained unclear. The results of a meta-analysis carried out by Bakouei et al. published in 2020 showed that omega-3 fatty acids had a protective role in the occurrence of preeclampsia in low-risk pregnancies, in the case of women who receive DHA + EPA, and who start supplementation from the second half of pregnancy. No protective effects were observed for preeclampsia in the case of high-risk pregnancies, supplementation only with DHA, and with first-half pregnancy supplementation [86].

## 7. Effects on Postpartum Depression

Postpartum depression is a global health problem, and it is more common in primiparous women. Depressive symptoms affect the quality of childrearing and could alter the bond between mother and child. Most of the time, mothers refuse treatment in order not to have negative effects on their children. The relationship between postpartum depression and the intake of omega-3 fatty acids was evaluated. The results of the study conducted by Harauma et al. indicated that the intake of α-linolenic acid during pregnancy improves mental health at one month after birth, and high concentrations of it in maternal erythrocytes were associated with a low Edinburgh Postnatal Depression Scale score (<9). The intake of omega-3 fatty acids did not show significant effects on Mother-to-Infant Bonding Scale scores [87].

It is well known that omega-3 fatty acids have an anti-inflammatory role. An increased ratio between omega-6 and omega-3 fatty acids could cause neuroinflammation, and many psychiatric conditions can be associated with them [88].

It was proved that omega-3 fatty acids play an important role in the occurrence of numerous nervous system disorders, including depression. DHA deficiency has been associated with a series of neuronal and signaling changes, such as neuronal membrane instability and negative effects on the transmission of dopamine and serotonin. All these result in the symptoms associated with depression [89]. The plasma concentration of DHA was lower in the case of women with postpartum depression than those without depressive symptoms. Alteration of DHA status increases the risk of depression, as in the case of women with a short period between two pregnancies [90].

Mothers who had an increased risk of postpartum depression have been shown to experience during pregnancy and breastfeeding a rapid depletion of omega-3 fatty acids. The supplementation with fish oil rich in EPA, both during pregnancy and during breastfeeding, reduced the symptoms of depression. At the same time, supplementation with DHA could reduce the risk of postpartum depression [91]. Supplements with a high EPA to DHA ratio (>1.5) had significant beneficial effects in mild to moderate pregnancy and postpartum depression [92]. Considering the published data for outside the perinatal period, supplements rich in EPA (2.2 g/day) can be used for major episodes of postpartum depression [93].

Contradictory data were reported in the literature regarding the benefits of DHA and EPA intake for preventing or reducing symptoms of depression. In a study conducted by Vas et al., the intake of 1.8 g of omega-3 fatty acids for 16 weeks had no effect on maternal depression [94].

## 8. The Effects of Supplementation with DHA and EPA during Breastfeeding

More than 200 fatty acids were identified in breast milk, of which the most important were DHA and EPA. The presence of DHA in breast milk was essential for optimal long-term reduction of the risk of chronic diseases [95] but also for growth, language, and neurological development [96]. Arachidonic acid (AA) has the highest percentage of the total long-chain polyunsaturated fatty acids in breast milk. AA has a very important role in the infant’s nutrition and development [97]. Arachidonic acid is very important for brain growth through its involvement in cell division and signaling processes. It was found that the level of arachidonic acid in breast milk is relatively stable [98].

It was discovered that DHA levels in breast milk had effects on visual acuity [69,99]. It was found that the administration of 200 mg of DHA in the first 4 months of postpartum breastfeeding had an impact on the neurodevelopment of children through beneficial effects on the psychomotor development at 30-month-old children [100], and improvements were observed in terms of the sustained attention of five-year-olds children [101]. Another study that evaluated the effects of supplementation in the first 4 months of breastfeeding women with fish oil (4.5 g) reported that there was no benefit in terms of problem resolution [102].

EPA concentration in breast milk was associated with maternal EPA status, which was closely related to EPA intake. It was found that breast milk rich in EPA had beneficial effects on infant distractibility, which is an early sign of Attention-Deficit/Hyperactivity Disorder (ADHD) [103].

In a study by Giuffrida et al. showed that alpha-linoleic acid is found in a proportion of 0.8–0.9% of the total fatty acids in breast milk. DHA in breast milk decreases from 0.68% on day 0 to 0.34% of total fatty acids in day 120 lactations, while the proportion of EPA is lower (0.12% of total fatty acids) and remains stable throughout lactation [104].

## 9. Other Effects on Pregnant Women

In addition to the beneficial effects on the fetus and infant, DHA was also found to be important for the mother’s health and was associated with a decrease in postpartum depression and cardiovascular risk [105]. It was observed that omega-3 fatty acids have an anti-inflammatory role and adjust the immune system, an antiarrhythmic role and adjust the metabolism of the myocardium, and a role in endothelial functions [106]. Both DHA and EPA derivatives (maresin, protectins, and resolvin D1), as well as their precursors, have a role in reducing the inflammatory reaction in pregnancy. It was observed that this effect is more obvious in the last trimester of pregnancy [107].

Protection against metabolic disease was another role of omega-3 fatty acids. Obesity was accompanied by a chronic inflammatory status. Thus, it was observed that the increased intake of omega-3 fatty acids can be beneficial in the management of obesity. Young women were likely to gain weight easily and to develop metabolic diseases (e.g., polycystic ovary syndrome associated with insulin resistance), which increased their vulnerability to omega-3 fatty acid deficiency [108].

Supplementation with omega-3 fatty acids has been observed to increase fertility and women’s likelihood of conceiving [109]. In addition, it was observed that the increased intake of omega-3 fatty acids could reduce the activity of autoimmune diseases such as rheumatoid arthritis, type 1 diabetes, and systemic lupus erythematosus [110].

## 10. Recommendations

Table 2 contains the recommendations of several specialized societies from around the world regarding the intake of DHA and EPA during pregnancy.

## 11. Conclusions

Docosahexaenoic acid and eicosapentaenoic acid are essential fatty acids for the human body, and their main source is the diet (i.e., they are rich in seafood) and supplements. It was observed that supplementation with DHA and EPA during pregnancy had beneficial effects on the neurological development of the fetus and infant by improving language, memory, attention, and hand coordination, affecting sleep patterns, and improving visual acuity. Supplementation with DHA and EPA could reduce the risk of preterm birth but also of preeclampsia in low-risk pregnancies. Beneficial effects on the mother have been identified, such as the reduction of postpartum depression symptoms, the reduction of cardiovascular risk, and the anti-inflammatory role.

However, the data in the literature are contradictory regarding the positive effects of omega-3 supplementation in pregnancy. Future studies are needed to evaluate the beneficial effects listed above.

## Figures and Tables

**Table 2:** The recommendations for the intake of DHA and EPA during pregnancy.

Society (Year)	Dose (Per Day)
Food and Agriculture Organization of the United Nations (2010) [111]	300 mg DHA + EPA;
	Of which at least 200 mg DHA
European Food Safety Authority (2010) [112]	250 mg DHA + EPA for all adults
	Additional 100–200 mg DHA in pregnancy
World Association of Perinatal Medicine (2008) [113]	200 mg DHA

## Data Availability

Data sharing is not applicable to this article.
